# Immune mechanisms of group B coxsackievirus induced viral myocarditis

**DOI:** 10.1080/21505594.2023.2180951

**Published:** 2023-02-24

**Authors:** Yue Zhang, Xiaobin Zhou, Shuyi Chen, Xinchen Sun, Chenglin Zhou

**Affiliations:** a Clinical Medical Laboratory Center, The Affiliated Taizhou People’s Hospital of Nanjing Medical University, Taizhou, China; b School of public health, Nantong University, Nantong, China

**Keywords:** Viral myocarditis, innate immunity, adaptive immunity, pattern recognition receptors (PRRs), T lymphocytes

## Abstract

Viral myocarditis is known to be a primary cause of dilated cardiomyopathy (DCM) that can lead to heart failure and sudden cardiac death and is invariably caused by myocardial viral infection following active inflammatory destruction of the myocardium. Although acute viral myocarditis frequently recovers on its own, current chronic myocarditis therapies are unsatisfactory, where the persistence of viral or immunological insults to the heart may play a role. Cellular and mouse experimental models that utilized the most prevalent Coxsackievirus group B type 3 (CVB3) virus infection causing myocarditis have illustrated the pathophysiology of viral myocarditis. In this review, immunological insights into the different stages of development of viral myocarditis were discussed, concentrating on the mechanisms of innate and adaptive immunity in the development of CVB3-induced myocarditis.

## Introduction

Viral myocarditis refers to the occurrence of inflammatory lesions in the myocardial tissue due to viral infection [1]. Although people of all ages, races, and genders are susceptible to contracting viral myocarditis, with the incidence varies from 10 to 22 per 100,000 people, this disease is most prevalent among adolescents and is quickly overtaking heart attacks as the primary cause of sudden cardiac death in youth [2]. Patients with viral myocarditis have a good chance of fully recovering; nonetheless, up to 20% of those infected will develop dilated cardiomyopathy (DCM) and heart failure [3–5]. Myocarditis spurred on by a virus had a 19.2% mortality over 4.7 y, with 9.9% of those deaths being caused by heart collapse [6].

Viral myocarditis progression is greatly influenced by immune responses [7]. Generally, viruses such as Coxsackievirus and Erythrovirus induce the production of cytokines following tissue damage, which is then followed by the infiltration of immune cells via concomitant pro-inflammatory and pro-fibrotic cytokine generation [8–10]. The progression of viral myocarditis may be roughly thought of as having three distinct stages. The primary stage is viral myocarditis, where the virus enters the host and is transported to the myocardium, thereby activating innate immunity [11]. The second stage is autoimmune myocarditis, in which adaptive immunity is triggered [12]. The third stage of viral myocarditis, distinguished by persistent inflammation and DCM, enhances if the virus is not entirely eliminated in the previous two stages [13]. Accordingly, the present review attempts to outline the research status pertaining to the relevant immune mechanisms of viral myocarditis based on the three developmental stages.

## Triggers of myocarditis

**Figure 1.**
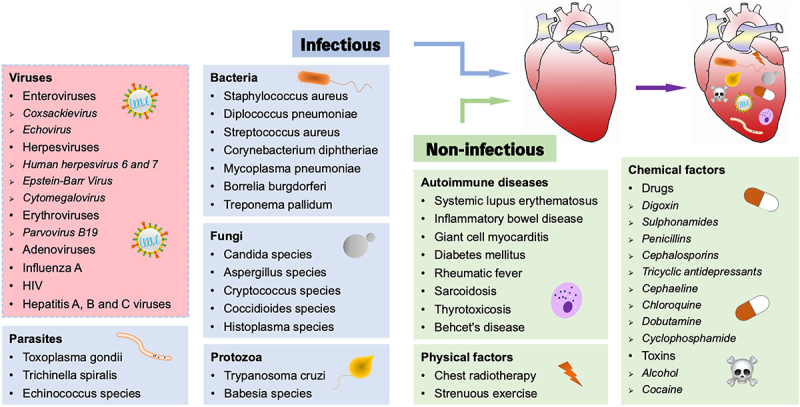
Triggers of myocarditis. Myocarditis can be induced by both infectious and non-infectious pathogens, with viral infection being the most common cause (red background).

Infectious pathogens, such as viruses and bacteria, as well as non-infectious elements including autoimmune diseases and chemical factors, can trigger myocarditis (Figure 1). Among them, viral infection is considered to be the most common cause of myocarditis, accounting for 50–70% of all cases in developed countries such as the United States and Europe [14,15]. Enteroviruses, especially Coxsackievirus, Herpesviruses, Erythroviruses, Adenoviruses, Influenza A, HIV, and Hepatitis viruses have received considerable research attention [16,17]. Coxsackievirus group B type 3 (CVB3) is a non-enveloped, positive-sense, single-stranded RNA virus belonging to the Enterovirus family [18]. CVB3 is spherical in shape, 22–30 nm in diameter, with a genome length of 7.4 kb. The nucleocapsid of CVB3 consists of four proteins, VP1, VP2, VP3, and VP4, which infect host cells by binding to (coxsackievirus and adenovirus receptor) CAR/CD55/(decay accelerating factor) DAF on the host cell membrane, resulting in the exposure of VP4 and VP1 proteins hidden inside the nucleocapsid to the viral surface and facilitating the wrapping and absorption of CVB3 by the host cell membrane [19,20]. There is substantial evidence that young adults and children are more vulnerable to CVB3-induced myocarditis. Furthermore, CVB3 is known to be the most extensively studied virus in both patients and animal models with myocarditis [21]. Over the past two decades, human herpesvirus 6 (HHV-6) has been associated with paediatric cardiomyopathy and DCM [22]. Parvovirus B19 (PVB19), a member of Erythroviruses, has also been reported in the paediatric population, which may lead to heart failure with high mortality [23]. Notably, HHV-6 and PVB19 have increasingly supplanted Coxsackievirus and Adenoviruses as the most frequently recognized viruses in viral myocarditis and DCM. Influenza A is an RNA virus belonging to the Orthomyxoviridae family that can evolve to myocarditis, where patients may manifest with palpitation, fever, shortness of breath, myalgia, and collapse [24]. Recent studies have demonstrated that severe acute respiratory syndrome coronavirus 2, which causes Coronavirus disease-19, may also give rise to myocarditis [25,26]. Thus, in order to study the pathogenesis of viral myocarditis, a model of CVB3 infection is usually used.

## Stages of CVB3-induced myocarditis

**Figure 2.**
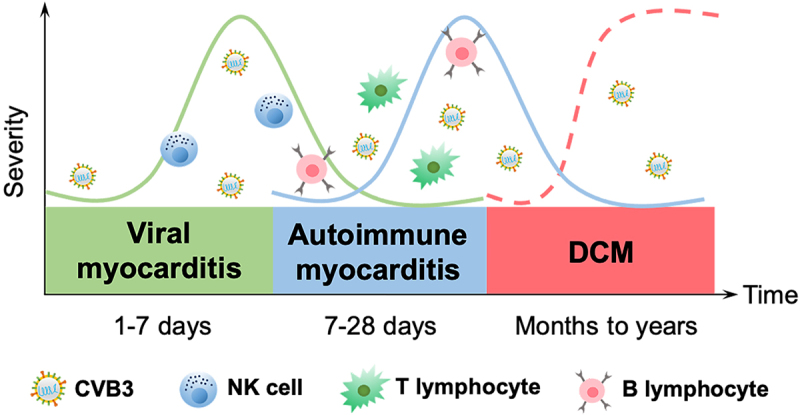
Temporal stages of CVB3-induced myocarditis. The first stage occurs when the virus enters into the host and translocates to the myocardium, causing various cellular responses and activating the host’s innate immunity for 1–7 d (green solid curve). The cellular and humoral responses lead to autoimmune-mediated damage in the second stage, with T lymphocyte infiltration cresting at 7–14 d, which typically lasts 7–28 d (blue solid curve). In certain patients, the inflammation fades as myocardial damage diminishes; nevertheless, in others, the virus stays in the body for months or years, giving rise to chronic inflammation and DCM (the third stage, red dotted curve).

CVB3-induced myocarditis can be classified into three stages according to its pathogenesis: viral myocarditis, autoimmune myocarditis, and DCM. During the viral myocarditis stage, CVB3 enters into myocytes and activates the host’s innate immunity, which is usually self-limiting. In autoimmune myocarditis, CVB3 enters into acute injured myocytes in order to replicate and induce myocardial infarction, thereafter activating adaptive immunity, which can be life-threatening [27]. Persistent inflammation of the myocardium leads to myocardial remodelling, which eventually develops into DCM and may give rise to cardiac fibrosis and trigger a decline in cardiac function, heart failure, and even death (Figure 2). As numerous immune cells and molecules are involved in each stage of viral myocarditis, the immune mechanisms of CVB3-induced myocarditis at different stages will be, respectively, explained.

### Viral myocarditis stage

Innate immunity is dominant during the viral myocarditis stage, in which the host activates innate immune cells via recognition of pathogen-associated molecular patterns (PAMPs) and damage-associated molecular patterns (DAMPs) through pattern recognition receptors (PRRs) following viral infection [28]. Among them, PRRs (Toll-like, NOD-like, and AGE receptors) activate immune cells via signal transduction in order to produce and secrete pro-inflammatory and antiviral cytokines, while natural killer (NK) cells are directly involved in the killing of virally infected cells and secreting cytokines through PRRs, as well as their surface-specific receptors, natural killer cell receptors [29–32].

#### Toll-like receptors (TLRs)

TLRs belong to membrane-bound PRRs, in which TLR3, TLR7, TLR8, and TLR9 recognize viral nucleic acid, while TLR2/4 recognize viral envelope protein [33,34]. A majority of TLRs activate myeloid differentiation factor 88 (MyD88)-related pathways, whereas others, including TLR3 rely on the TIR-domain-containing adapter-inducing interferon-β (TRIF) pathway [35–37]. Specifically, TLR4 can simultaneously initiate both the MyD88 and TRIF pathways.

TLR3, which is mainly expressed on the endosomal membrane, induces the expression of antiviral cytokine IFN-γ [38]. Studies on adiponectin^−/−^ and wild-type (WT) mice have shown that CVB3 can trigger innate immunity by combining the CD14-TLR complex. Increased expression of CD14 and proinflammatory cytokines has also been seen in adiponectin-deficient mice infected with CVB3. In addition, incubation with TLR3 agonists has been reported to stimulate IFN expression in cardiomyocytes, whereas the addition of adiponectin has been shown to suppress this production [39]. These results indicate the importance of adiponectin in promoting the progression of viral myocarditis via inhibition of the TLR3-mediated innate immune response. Studies on protease activated receptor (PAR)2 and viral myocarditis have shown that IFN-β expression and signal transducer and activator of transcription (STAT)1 phosphorylation are elevated in PAR2^−/−^ cardiac fibroblasts (CFs) compared to that of PAR2^+/+^ CFs after CVB3 infection. Meanwhile, STAT1 phosphorylation of PAR2 overexpressed CFs has been shown to be significantly decreased following PAR2 and TLR3 agonist stimulation, suggesting that PAR2 negatively regulates TLR3-dependent IFN-β production and promotes viral myocarditis [40]. Antoniak et al. found that PAR1^−/−^ mice expressed reduced levels of IFN-β and C-X-C motif chemokine ligand (CXCL)10 compared to that of PAR1^+/+^ mice, while PAR1 and TLR3 co-enhanced phosphorylation of p38 and production of IFN-β and CXCL10 in mice CFs. These findings suggest that PAR1 positively regulates the production of IFN-β and CXCL10 through TLR3-p38 in order to improve viral myocarditis [41]. From what we can gather from the aforementioned research, TLR3 is primarily responsible for mediating IFN production and exhibits an antiviral role in viral myocarditis.

TLR4 is abundantly found in the human heart and is primarily membrane-expressed [42]. Recent studies have found that CVB3 infection upregulates TLR4 expression on the cell membrane [43]. As proposed by Rienks et al., a novel 72-kDa osteoglycin (iOGN) was expressed on circulating innate immune cells and cardiac myocytes, and iOGN co-immunoprecipitated with TLR4 in lysates of human peripheral leukocytes and bone marrow-derived macrophages (BMDMs). Following LPS stimulation, the expression levels of MyD88, p-PI3K, and p-cjun in iOGN^−/−^ BMDMs from mice have also been shown to be lower than that in iOGN^+/+^ BMDMs, indicating that iOGN on innate immune cells promotes the development of viral myocarditis through the activation of the TLR4-MyD88-mitogen-activated protein kinase (MAPK) pathway [44].

TLR7 is mostly expressed on the endoplasmic reticulum membrane [45]. Moreover, interleukin-1 receptor-associated kinase 4 (IRAK4), which is a downstream molecule of MyD88, plays an important role in pro-inflammatory cytokine production for the stimulation of TLR2, TLR4, TLR7/8, TLR9 in macrophages and myeloid dendritic cells [46]. Valaperti et al. found that after CVB3 infection, TLR7-stimulated IRAK4^−/−^ mice exhibited significantly upregulated STAT5 phosphorylation and enhanced IFN-α and IFN-β expression compared with that of IRAK4^+/+^ mice, indicating that IRAK4 accelerates viral myocarditis progression by inhibiting TLR7-mediated IFN production [47].

TLR8 is mainly expressed on the endoplasmic reticulum membrane of myeloid dendritic cells, monocytes/macrophages, and neutrophils [48]. Rivadeneyra et al. reported that human neutrophils internalized and recognized CVB3 through endosomal TLR8 and then triggered NF-κB signalling, resulting in the upregulation of CD11b, strengthening of the adhesion to fibrinogen and fibronectin, and increase in secretion of IL-6, IL-1β, TNF-α, and IL-8, which suggests that neutrophils inhibit viral myocarditis development through TLR8 [49].

MyD88 is a protein that plays a prominent part in the conventional TLR signal transduction pathway, activating transcription factors NF-κB and AP-1 via protein kinases [50]. Shi et al. found that CVB3 cleaved sequestosome 1 (SQSTM1, p62) through viral protease 2A^pro^, making it unable to interact with ubiquitinated proteins and disrupting selective autophagy. Furthermore, SQSTM1 has been shown to lose the ability to activate NF-κB signalling, leading to the further development of viral myocarditis [51].

In contrast to majority of TLRs, which utilize the MyD88 pathway to transduce signals, fractional TLRs utilize MyD88-independent pathway, namely the TRIF pathway, which commences with TRIF activation of IRF-3 and IRF-7 and culminates in IFN-I production [52]. Liu et al. investigated a tripartite motif-containing (TRIM)21 and found that TRIM21 expression in the heart of CVB3-infected mice was upregulated, while mRNA expression of IFN-α and IFN-β was shown to be significantly increased in TRIM21-overexpressed cells infected with CVB3, which was promoted by IRF3 dimerization and phosphorylation [53]. The corresponding findings suggest that TRIM21 positively regulates the IFN-I pathway via promotion of IRF3 activation and inhibition of viral myocarditis progression.

#### NOD-like receptors (NLRs)

NLRs, which consist of C-terminal leucine-rich repeat, nucleotide-binding domain, and N-terminal effector domain, are located in the cytosol and act as intracellular PRRs [54]. According to the N-terminal effector domain, NLRs are classified into five subfamilies: NLRA, NLRB, NLRC, NLRP, and NLRX. A part of NLRs, in conjunction with the adaptor protein apoptosis-associated speck-like protein containing a CARD (ASC) and effector caspase-1, form the inflammasome and initiate the inflammatory response [55].

Wang et al. found that the expression of IL-1β, as well as the inflammasome components ASC and caspase-1, is upregulated in the cardiac tissue of CVB3-infected mice. Meanwhile, IL-1β level was shown to be downregulated with caspase-1 inhibitor, while symptoms of mice myocarditis were observed to be alleviated following the use of neutralizing antibody against IL-1β, which suggests that CVB3-induced inflammasome activation plays a role in viral myocarditis through the regulation of IL-1β production. Meanwhile, they also found that potassium efflux and reactive oxygen species (ROS) were indispensable for NLRP3 inflammasome activation [56]. In addition, Bao et al. demonstrated that the expression of NLRP3 and IL-1β was upregulated in the cardiac infiltrating macrophages of CVB3-infected mice. Here, CVB3 is shown to trigger macrophage NLRP3 activation and upregulation with CVB3 capsid viral proteins VP1 and VP2 instead of viral RNAs [57].

#### AGE receptors (RAGEs)

RAGE is a distinct type of PRR implicated in a variety of pathological processes, which also regulates inflammatory cell migration and oxidative stress [58].

Muller et al. discovered that CVB3-infected mice had increased S100 calcium-binding protein (S100)A8 and S100A9 expression and myocardial dysfunction; however, CVB3-infected S100A9^−/−^ mice exhibited decreased RAGE and diaphanous-1 (Dia-1) adaptor mRNA expression compared with that of CVB3-infected WT mice. Meanwhile, the myocardial function of CVB3-infected S100A9^−/−^ mice had a similar recovery process to WT mice when supplemented with S100A8. These findings suggest that CVB3 infection leads to the elevation of DAMPs S100A8 and S100A9 expression, which enhances the inflammatory response via RAGE/Dia-1 pathway [59].

#### NK cell receptors (NCRs)

NCRs, including NKp46, NKp30, and NKp44, constitutively expressed on NK cells, activate cytolytic functions of NK cells in viral clearance and host immunity [60,61]. The transcription factor forkhead box (Fox)o3 has been described to be involved in cell metabolism, oxidative stress, and inflammatory disease [62]. Loebel et al. found that CVB3-infected Foxo3^−/−^ mice had increased expression of IFN-γ and NKp46 as well as a higher proportion of CD11b^+^CD27^+^ NK cells. Moreover, they were found to have enhanced CD69 expression on NK cells with higher cytotoxicity, suggesting that Foxo3 plays a critical role in innate immunity through the regulation of NK cell function by NKp46 in CVB3 infection [63].

### Autoimmune myocarditis stage

The autoimmune myocarditis stage is strongly influenced by adaptive immunity. Due to the cytolytic properties of CVB3 virus, intracellular and surface antigens are released from the heart, where newly released antigens activate T and B lymphocytes, triggering autoimmune responses. In this stage, antigen-presenting cells (APCs) represent the viral or host antigens and activate CD4^+^ T cells in order to differentiate into Th cell subsets while secreting cytokines [64]. Th cells then assist B cells in secreting specific antibodies to neutralize the virus or the host antigens with co-stimulatory molecules and cytokines while activating CD8^+^ T cells to differentiate into cytotoxic T lymphocytes (CTLs) to kill virally infected cells [65].

#### CD4^+^ T cells

CD4^+^ T cells are activated by the complex formed by antigenic peptides and MHC class II molecules, which are mainly differentiated into four subsets: Th1, Th2, Th17, and Treg. Th1 and Th17 cells mostly play pro-inflammatory roles, while Th2 and Treg cells have predominantly anti-inflammatory roles. Multiple investigations have confirmed that the proportion and differentiation of CD4^+^ Th cells are closely related to the development of viral myocarditis [66].

##### Th1 and Th17 cells

The hallmark cytokine produced by Th1 cells is IFN-γ. The transcription factor involved in Th1 cell differentiation has been shown to be T-bet, in which STAT1 and STAT4 regulate the secretion of IFN-γ and IL-12, respectively. Meanwhile, the hallmark cytokine produced by Th17 cells is IL-17, while the specific transcription factor of Th17 cells is retinoic acid receptor-related orphan receptor γ (RORγT), where STAT3 induces RORγT expression [67,68].

Long et al. illustrated that CVB3 entered into CD4^+^ T cells and showed that Th17 cell proportion, IL-17 secretion, and RORγT synthesis increased after purified CD4^+^ T cells were transfected with CVB3 [69]. Meanwhile, Yuan et al. discovered that in mice infected with CVB3, the frequency of splenic Th17 cells, IL-17 mRNA, RORγT, and cardiac CVB3 RNA were all increased. However, CVB3 RNA was shown to be suppressed following the neutralization of IL-17 [70]. The above studies suggest that IL-17 produced by Th17 cells promotes CVB3 replication and augments the severity of viral myocarditis.

Wei et al. transferred purified IL-10^+^ B cells from WT mice and CVB3-infected mice into B cell-deficient mice with viral myocarditis, demonstrating that splenic Th1 and Th17 cell proportions, cardiac T-bet, and RORγT mRNA from CVB3-infected mice were significantly lower than those from WT mice. These findings suggest that IL-10^+^ B cells downregulate the proportions of Th1 and Th17 cells in CVB3-infected mice [71]. Li et al. showed that plasma and cardiac progranulin were increased in CVB3-infected WT mice, and viral myocarditis was exacerbated in CVB3-infected progranulin^−/−^ mice with high levels of splenic and cardiac Th1 and Th17 cell proportion, which were also evident in serum levels of IFN-γ, TNF-α, IL-17A, and IL-21. Moreover, p-Janus kinases (JAK)2, p-STAT4, p-JAK3, and p-STAT3 were found to be decreased when purified mouse CD4^+^ T cells were co-incubated with progranulin, indicating that progranulin inhibits Th1 and Th17 cell differentiation via JAK/STAT pathway in viral myocarditis [72].

##### Th2 and Treg cells

The specific transcription factors of Th2 and Treg cells are GATA binding protein 3 (GATA3) and Foxp3, respectively, where STAT6 serves as an essential component in Th2 differentiation, and STAT5 is vital in Treg signalling [68].

Zhou et al. activated the cholinergic anti-inflammatory pathway (CAP) in CVB3-infected mice and found that splenic GATA3 and Foxp3 expression was induced, while that of T-bet and RORγT was reduced. Meanwhile, Th2 and Treg cell proportions were shown to be increased, Th1 and Th17 cell proportions were decreased, and the survival rate of CVB3-infected mice was elevated, demonstrating that CAP ameliorates viral myocarditis via regulation of Th cell differentiation into Th2 and Treg cells [73].

#### CD8^+^ T cells

IL-21 is a Th17-derived cytokine that signals via IL-21 R in order to mediate the activation, proliferation, and cytotoxic activity of CD8^+^ T cells [74]. Liu et al. found that the severity of CVB3-infected IL-21 R^−/−^ mice was significantly alleviated, in which the number of CD8^+^ IFN-γ^+^ T cells was found to be decreased, though the number of CD4^+^ T cells remained unchanged. CVB3-infected CD8^−/−^ mice had attenuated myocarditis symptoms when transferred with CD8^+^ T cells from WT mice instead of IL-21 R^−/−^ mice, suggesting that IL-21 R signalling regulates viral myocarditis via activation of CD8^+^ T cells [75].

#### B cells

Aside from their more well-known functions as the secretion of antibodies, B cells also play significant roles in antigen delivery and regulation of T cell immunity [76,77]. CVB3 virus and the host overlap antigenic epitopes, where B cells secrete antibodies against viral antigens, causing the cross-reactivity with autoantigens and leading to autoimmunity [78]. On the contrary, B cells also play roles in regulating Th cell differentiation in CVB3-induced myocarditis. Mice deficient in B cells that were subsequently infected with CVB3 were created by Cent et al. They found that Th1 and Th17 cell differentiation was suppressed, while Th2 cell differentiation was boosted and cardiac damage was minimized, implying that B cells have distinctive pathogenic functions in viral myocarditis independent of T cells [79]. Similarly, when Lu et al. eliminated B cells in CVB3-infected mice, they discovered a comparable reduction in the spleen, blood, and heart Treg cells, as well as a reduction in splenic Treg cell activation and immunological activity, and a reduction in myocardial TGF-β and Foxp3 transcript levels. This suggests that B cells, in addition to their pro-inflammatory activity in viral myocarditis, also play crucial roles in maintaining Treg cell homoeostasis [80]. Additionally, a study by Li et al. found that knocking out B cells in CVB3-infected mice lowered myocardial case scores and increased the M2 macrophage proportion, whereas after reconstitution of B cells, the M2 macrophage proportion decreased, indicating that B cells promote viral myocarditis progression by inhibiting M2 polarization [81].

### DCM stage

Incomplete CVB3 clearance and persistent chronic inflammation may lead to DCM, which is characterized by fibrosis and cardiac remodelling [82]. Cardiac macrophages and fibroblasts produce IL-6, which is crucial for myocarditis exacerbating into DCM, while IL-6 inhibition reduces angiotensin II-induced cardiac fibrosis [83]. Kraft et al. found that in CVB3-infected mice, the expression levels of extracellular regulated protein kinase (ERK)1/2, extracellular matrix (ECM), and fibrosis-related molecules such as TGF-β and matrix metalloproteinase (MMP)12 were increased; however, the expression of ERK1/2, IL-6 and the above molecules were noted to be decreased when treated with IL-1β neutralizing antibody in the mouse model. Furthermore, cardiac injury and fibrosis were mitigated, suggesting that IL-1β influences the expression of ECM and fibrosis-related molecules, cardiac remodelling and DCM via regulation of ERK1/2 and IL-6 [84]. Liu et al. induced DCM mice with CVB3 and found that the expression of pro-inflammatory cytokines IL-1β, IL-6, TNF-α, IFN-γ was upregulated in the hearts of DCM mice, with cardiac fibrosis being aggravated. Moreover, CVB3-induced expression of these cytokines was suppressed in mice treated with astragalus polysaccharide, which played a protective role against cardiac fibrosis [85]. Upregulation of IL-6 mRNA levels in myocardial tissues was observed in DCM mice, as reported by Li et al. Following IL-6 knockdown in DCM mice, myocardial apoptosis and cardiac remodelling were suppressed, as was the production of fibrosis indicators such as collagen type (COL)1-A1 and collagen type (COL)III-A1. This was consistent with a reduction in STAT3 phosphorylation, demonstrating that IL-6-mediated STAT3 signalling pathway induces myocardial apoptosis and cardiac remodelling [86]. Furthermore, Li et al. found that human cardiac fibroblasts produced large amounts of IL-6 and IL-8 with stimulation of TLR2 and TLR4, and that cardiac infiltrated immune cells show an uncontrolled response, which further deteriorated cardiac function, damaged cardiac tissues and caused DCM [87]. A study by Guo et al. further demonstrated that CVB3-induced DCM was protected by IL-22-producing Th22 cells, illustrating that splenic Th22 cell numbers, plasma IL-22 levels, and myocardial IL-22 R expression were all elevated in the mice with DCM. While a neutralizing antibody against IL-22 led to increased COL1-A1, COL3-A1, MMP9 expression, and intensified myocardial fibrosis in DCM mice, suggesting that IL-22 protects the myocardium by inhibiting myocardial fibrosis [88].

## Conclusions and remarks

**Figure 3.**
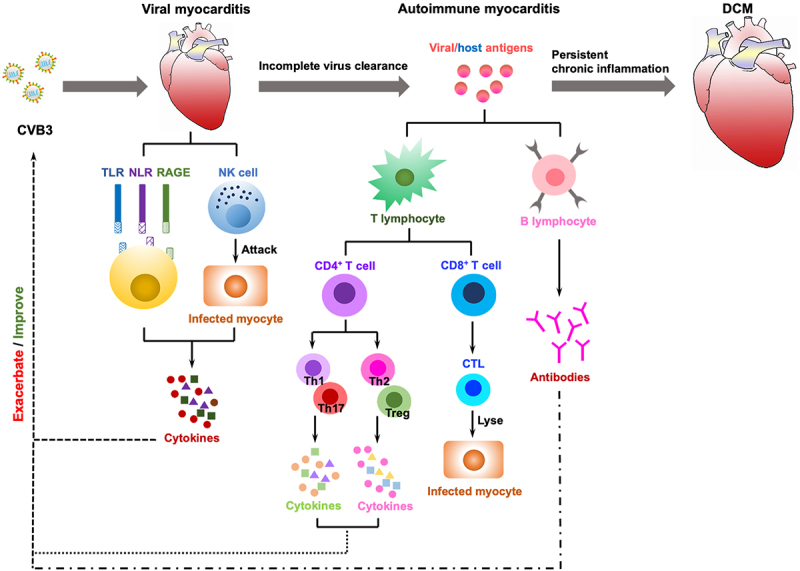
The immune mechanisms in the development of CVB3-induced myocarditis. Viral myocarditis is attributed to a combination of viral and host factors in vulnerable people. As viruses replicate, they trigger a cascade of PRRs (TLRs, NLRs, RAGEs) that are essential for activating immune cells and mediating the production of cytokines; simultaneously, NK cells directly destroy virally infected cells. In some cases, the virus will be eradicated during this process; otherwise, it can progress to chronic myocarditis or DCM with adaptive immunity engaged. The release of viral antigens is then followed by delivery through APCs (e.g. dendritic cells) that activate CD4^+^ T cells to develop into distinct subsets and secrete cytokines, whereas CD8^+^ T cells differentiate into CTLs that lyse virally infected cells. B cells secrete antibodies, and with the induction of anti-viral T cells and antibody responses, the infectious virus may be eradicated. After viral lysis of the infected cells, intracellular proteins (i.e. cardiac myosin) or cryptic epitopes (i.e. host antigens), are released and presented by APCs to CD4^+^ T cells, CD8^+^ T cells and B cells, which mediate inflammation through the secretion of cytokines, cytolysis, and production of autoantibodies, respectively, triggering an autoimmune response that in turn infiltrates the heart and exacerbates inflammation. Eventually, if the virus clears, the myocardium can revert to normal, but delayed or ineffective viral clearance generates myocyte degeneration, interstitial fibrosis, hypertrophy, and DCM.

The most prevalent cause of viral myocarditis is viral infection, which is typically self-limiting but can develop into autoimmune disorders that may be life-threatening in extreme cases, particularly during young adulthood. CVB3 is a cytolytic virus that may induce cardiomyocyte damage, while persistent CVB3 viral infections and autoimmune damage can lead to chronic myocarditis and DCM. A variety of immune cells, receptors, and cytokines are involved in the evolution of the response from self-limiting viral myocarditis to autoimmune myocarditis and ultimately to inflammatory DCM, and innate cytokines can boost CVB3 (Figure 3). Innate immunity is dominant in the viral myocarditis stage, with PRRs, such as TLRs, NLRs, and RAGEs, engaged in the signalling, activation of immune cells and secretion of cytokines. TLRs contribute to innate immunity via various signalling pathways, such as MyD88-dependent TLR4-MyD88-MAPK signalling and MyD88-independent TRIF-IRF3 signalling, and secrete cytokines including IFN, IL-1β, IL-6, TNF-α. Meanwhile, NLRs and RAGEs both play roles in the viral myocarditis through the NLRP3 inflammasome and RAGE/Dia pathways, respectively. In addition, NK cells directly kill infected cells and have a role in immune clearance, where they secrete cytokines. Adaptive immunity predominates in the autoimmune myocarditis stage, and CD4^+^ T cells differentiate into various Th subsets, among which Th1 and Th17 cell subsets secrete cytokines IL-17, IFN-γ, TNF-α, and IL-21 in order to promote viral myocarditis progression, whereas differentiation to Th2 and Treg cell subsets improves viral myocarditis. Meanwhile, activated CD8^+^ T cells can differentiate into CTLs that specifically kill virus-infected target cells, thereby alleviating viral myocarditis, while B cells secrete antibodies to neutralize the virus. Unfortunately, the low regenerative potential of cardiomyocytes makes full restoration of cardiac function unlikely even after antiviral immune responses or T cell activity have eliminated the virus. Simultaneously, the antigens produced as a result of viral damage may become autoimmune targets, leading to the triggering of pathogenic autoreactive T cells and antibodies, thus triggering an autoimmune response. Autoimmune myocarditis persists and progressively evolves into DCM with the involvement of cardiac macrophages, fibroblasts, and cytokines, in which cytokines such as IL-1β and IL-6 promote DCM at this stage. As the immunological mechanism of viral myocarditis continues to be explored, having a better understanding of the pathophysiology of viral myocarditis and generating novel ideas for clinical diagnosis and therapy can be helpful.

## Abbreviations

DCM: dilated cardiomyopathy
CVB3: Coxsackievirus group B type 3
CAR: coxsackievirus and adenovirus receptor
DAF: decay accelerating factor
HHV6: human herpesvirus 6
PVB19: Parvovirus B19
PAMPs: pathogen-associated molecular patterns
DAMPs: damage-associated molecular patterns
PRRs: pattern recognition receptors
NK: natural killer
TLRs: Toll-like receptors
NLRs: NOD-like receptors
RAGE: AGE receptors
NCRs: natural killer cell receptors
MyD88: myeloid differentiation factor 88
TRIF: TIR-domain-containing adapter-inducing interferon-β
IFN: interferon
WT: wild-type
PAR: protease activated receptor
STAT: signal transducer and activator of transcription
CFs: cardiac fibroblasts
CXCL: C-X-C motif chemokine ligand
iOGN: osteoglycin
BMDMs: bone marrow-derived macrophages
LPS: lipopolysaccharide
p-PI3K: phospho-phosphoinositide 3-kinase
NF-κB: nuclear factor kappa-B
MAPK: mitogen-activated protein kinase
IRAK4: interleukin-1 receptor-associated kinase 4
IL: interleukin
TNF: tumour necrosis factor
AP-1: activating protein-1
SQSTM1: sequestosome
IRF: interferon regulatory factor
TRIM: tripartite motif-containing
ASC: apoptosis-associated speck-like protein containing a CARD
ROS: reactive oxygen species
S100: S100 calcium binding protein
Dia-1: diaphanous-1
Fox: forkhead box
APCs: antigen-presenting cells
CTLs: cytotoxic T lymphocytes
RORγT: retinoic acid receptor-related orphan receptor γ
JAK: Janus kinases
GATA3: GATA binding protein 3
CAP: cholinergic anti-inflammatory pathway
TGF: transforming growth factor
ERK: extracellular regulated protein kinase
ECM: extracellular matrix
MMP: matrix metalloproteinase
COL: collagen type
